# Corticotropin-Releasing Factor Modulates Binge-Like Ethanol Drinking in a Sex-Dependent Manner: Impact of Amygdala Deletion and Inhibition of a Central Amygdala to Lateral Hypothalamus Circuit

**DOI:** 10.1016/j.bpsgos.2024.100405

**Published:** 2024-10-25

**Authors:** Sophie C. Bendrath, Hernán G. Méndez, Anne M. Dankert, Jose Manuel Lerma-Cabrera, Francisca Carvajal, Ana Paula S. Dornellas, Sophia Lee, Sofia Neira, Harold Haun, Eric Delpire, Montserrat Navarro, Thomas L. Kash, Todd E. Thiele

**Affiliations:** a Department of Psychology and Neuroscience, University of North Carolina at Chapel Hill, Chapel Hill, North Carolina; b Bowles Center for Alcohol Studies, University of North Carolina at Chapel Hill, Chapel Hill, North Carolina; c Department of Cell Biology and Physiology, University of North Carolina at Chapel Hill, Chapel Hill, North Carolina; d Department of Psychology, University of Almería, Almería, Spain; e Department of Pharmacology, University of North Carolina at Chapel Hill, Chapel Hill, North Carolina; f Department of Anesthesiology, Vanderbilt University Medical Center, Nashville, Tennessee

**Keywords:** Binge-like, Central amygdala, Chemogenetic, Corticotropin-releasing factor, Drinking in the dark, Ethanol, Lateral hypothalamus, Sex differences

## Abstract

**Background:**

Binge alcohol drinking is a dangerous behavior that can contribute to the development of more severe alcohol use disorder. Importantly, the rate and severity of alcohol use disorder has historically differed between men and women, suggesting that there may be sex differences in the central mechanisms that modulate alcohol (ethanol) consumption. Corticotropin-releasing factor (CRF) is a centrally expressed neuropeptide that has been implicated in the modulation of binge-like ethanol intake, and emerging data highlight sex differences in CRF systems.

**Methods:**

In the current report, we characterized CRF+ neurocircuitry arising from the central nucleus of the amygdala (CeA) and innervating the lateral hypothalamus (LH) in the modulation of binge-like ethanol intake in male and female mice.

**Results:**

Using chemogenetic tools, we found that silencing the CRF+ CeA to LH circuit significantly blunted binge-like ethanol intake in male but not female mice. Consistently, genetic deletion of CRF from neurons of the CeA blunted ethanol intake exclusively in male mice. Furthermore, pharmacological blockade of the CRF_1_ receptor in the LH significantly reduced binge-like ethanol intake in male mice only, while CRF_2_ receptor activation in the LH failed to alter ethanol intake in either sex. Finally, a history of binge-like ethanol drinking reduced *Crf* messenger RNA levels in the CeA regardless of sex.

**Conclusions:**

These observations provide novel evidence that CRF+ CeA to LH neurocircuitry is more sensitive for modulating binge-like ethanol intake in male mice, which may provide insight into the mechanisms that guide known sex differences in binge-like ethanol intake.

## Plain Language Summary

Binge alcohol drinking is a dangerous behavior that can contribute to more severe alcohol use disorders, and central corticotropin-releasing factor (CRF) signaling has been identified as an important modulator of binge drinking. Here, Bendrath *et al.* identify a CRF+ circuit between the central amygdala and lateral hypothalamus, and by employing a combination of pharmacological, chemogenetic, and genetic approaches, they show that this CRF+ circuit is more sensitive to modulating binge drinking in male than in female mice.

In the United States, alcohol (ethanol) binge drinking is a public health concern associated not only with many negative acute and long-term neuropsychological effects but also with the development of alcohol use disorder (AUD) (1, 2, 3, 4, 5). The National Institute on Alcohol Abuse and Alcoholism defines binge drinking as alcohol consumed within a 2-hour time frame that leads to blood ethanol concentrations (BECs) of ≥80 mg/dL (6). Males have been more often diagnosed with AUD than females; however, the numbers of AUD diagnoses in females have risen recently (7,8). Interestingly, there seem to be distinct differences between the male and female drinking populations such that a history of trauma and acute life stressors influences alcohol craving and relapse almost exclusively in females compared with males (9,10). This suggests an underlying common circuitry between stress and alcohol craving that may be sex specific but remains largely unknown.

Corticotropin-releasing factor (CRF) is a prostress peptide in the extended amygdala that becomes dysregulated during alcohol use and addiction (11). The central nucleus of the amygdala (CeA) is a particularly important region in relation to AUD, integrating stress and reward reactions to events to form behavioral responses (12). Previous work from our laboratory using drinking in the dark (DID) procedures to model binge-like ethanol intake in male mice has shown that 1-, 3-, and 4-day cycles of binge-like ethanol intake promote increased CRF protein expression in the CeA (13) as well as long-lasting subsequent increases of voluntary ethanol intake (14), suggesting extended plasticity stemming from a history of binge-like ethanol intake. During acute alcohol administration in rats, the CeA exhibits augmented release of the CRF peptide (15), which persists into withdrawal, and infusion of CRF into the CeA increases anxiety-like behavior during abstinence, suggesting that CRF may play a role in alcohol craving (16,17). Consistent with evidence of plasticity of central CRF systems in response to ethanol, we have found that CRF_1_ receptor (CRF1R) antagonists administered peripherally or into the CeA (13) and CRF_2_ receptor (CRF2R) agonists administered ventricularly (18) blunt binge-like ethanol intake in male mice. While these results implicate CRF1R inhibition and CRF2R activation in blunting binge-like ethanol intake, it is notable that a global genetic deletion study implicated the CRF1R as being primarily involved in modulating binge-like ethanol intake (19).

Notably, the CeA is the main output area of the amygdala and has strong projections to other prostress areas, such as the lateral hypothalamus (LH) (11,20). The LH is a heterogeneous brain area expressing a variety of neuropeptide systems and has been linked to a number of brain regions that modulate stress and reward (20). Stress-induced avoidance behavior in male rats as well as stress-induced anxiety-like behavior and ethanol self-administration in male and female rats are directly modulated by a CRF1R-positive (CRF1R+) neuronal circuit from the CeA to the LH, which functionally links these 2 brain areas (21,22). At the receptor level, LH *Crf1r* and *Crf2r* messenger RNA (mRNA) expression and ethanol consumption are positively correlated in male rats (23), and CRF1R in the LH modulates stress responses (24); thus, CRF1R and CRF2R in the LH may be potential targets for CRF projections from the CeA that modulate binge-like ethanol drinking.

Importantly, the role of CeA CRF+ neuronal innervation of the LH and CRF receptor signaling in the LH in the modulation of binge-like ethanol intake, as well as potential sex differences, are not well understood. With respect to the LH, evidence suggests that female rats are more likely to self-stimulate the LH than male rats (25), making this a sex-specific hedonic brain area. Within the extended amygdala, female mice lacking beta endorphin showed higher ethanol consumption and *Crf* mRNA in the extended amygdala than their wild-type control mice, phenotypes that were not evident in male mice (26). Additionally, compared with male rats, CRF receptor binding is increased in the cortex and amygdala of female rats (27), and *Crf* mRNA expression is greater in the amygdala of female mice (28). At the level of cell signaling, acute application of ethanol onto mouse CeA slice preparations reduced GABA (gamma-aminobutyric acid) release onto CRF1R-expressing neurons in male but not female mice, and application of exogenous CRF increased the firing rate of CRF1R-expressing neurons to a greater extent in male mice (29). Thus, while there is emerging evidence of sex differences in the sensitivity of the amygdala and hypothalamic systems and how they respond to ethanol, much more work is necessary to understand the mechanisms that are involved.

In light of the converging evidence that CRF signaling in the CeA modulates ethanol intake and our evidence here of a CRF+ neurocircuit arising from the CeA and innervating the LH, the current study was aimed at assessing the role of a CRF+ pathway from the CeA to the LH and CRF receptor signaling in the LH in the modulation of binge-like ethanol drinking. Additionally, experiments were conducted to investigate whether binge-like ethanol consumption alters *Crf* and *Crf* receptor mRNA in these brain regions and whether genetic deletion of CRF or CRF1R in the neurons of the CeA would impact binge-like ethanol intake. Importantly, because there has been very little investigation into potential sex differences in the mechanisms by which CRF signaling modulates binge-like ethanol intake, in vivo pharmacology, genetic deletion, chemogenetic studies, and quantitative polymerase chain reaction (qPCR) experiments described below included both male and female mice.

## Methods and Materials

See the Supplement for a detailed description of the procedures and statistical analyses.

## Results

### Gi-DREADD Silencing of the CeA to LH Pathways Revealed a Sex-Specific Reduction in Ethanol Drinking in Male Mice Only

**Figure 1:**
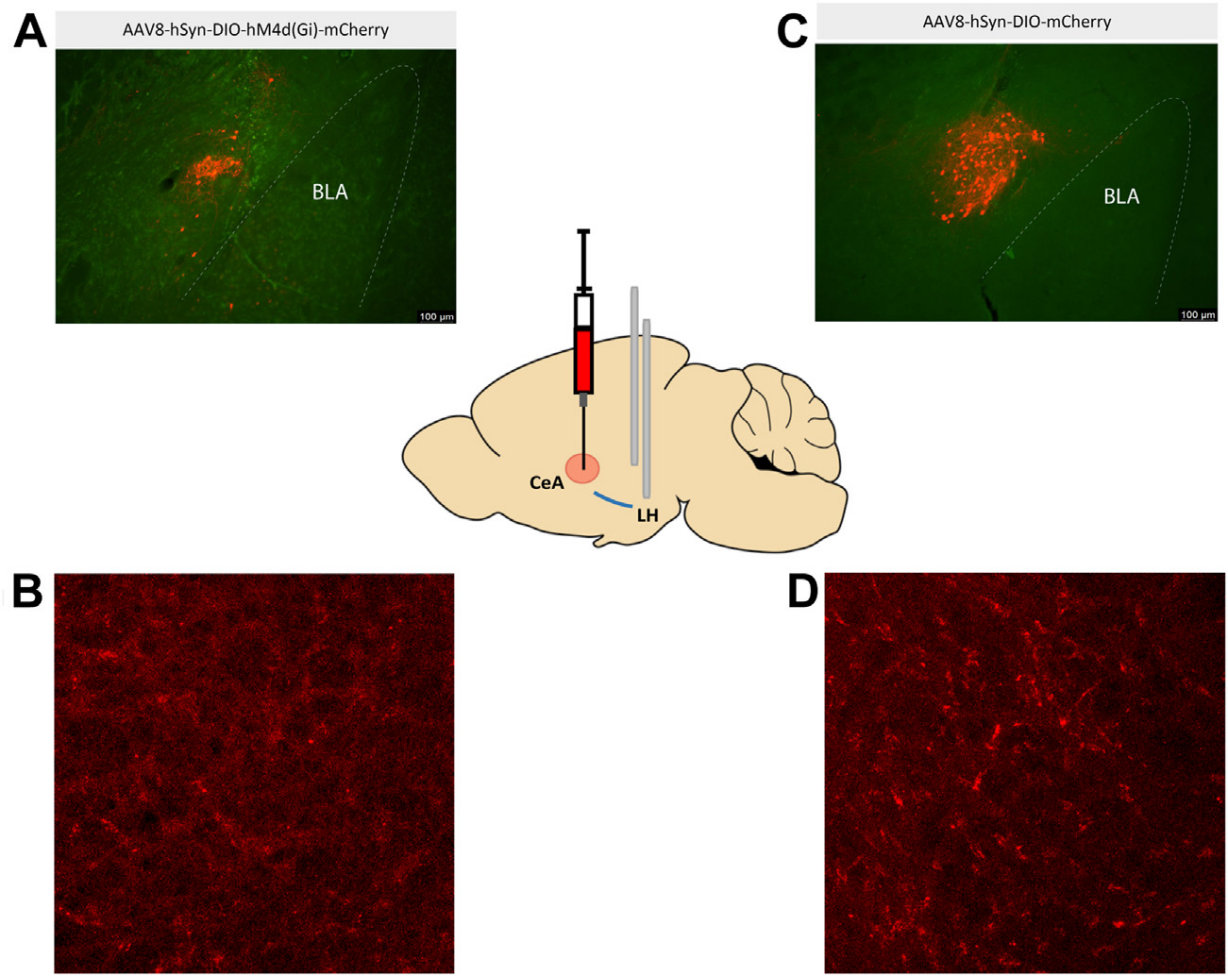
Photomicrographs showing AAV8-hSyn-DIO-hM4d(Gi)-mCherry virus expression in the CeA **(A)** and LH terminals **(B)**, and AAV8-hSyn-DIO-mCherry control virus expression in the CeA **(C)** and LH terminals **(D)** of CRH-ires-cre mice. The central schematic shows virus infusion into the CeA and cannalue placement into the LH. BLA, basolateral amygdala; CeA, central nucleus of the amygdala; LH, lateral hypothalamus.

**Figure 2:**
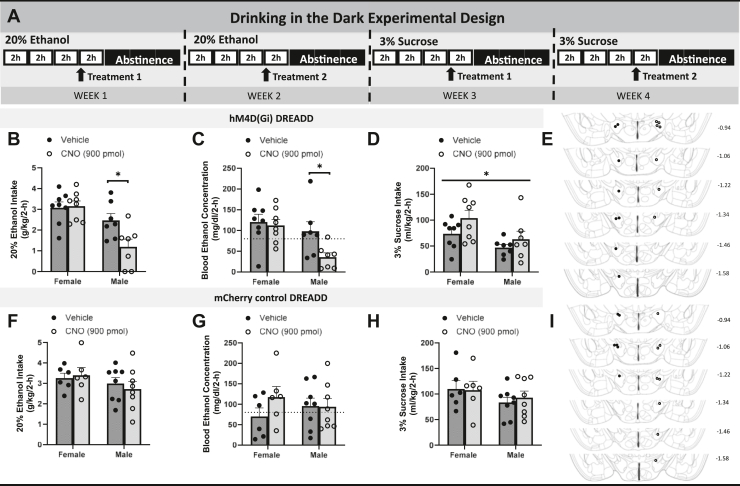
Chemogenetic silencing of the CRF+ CeA-LH circuit and control virus experiments. **(A)** Timeline of procedures used in the chemogenetic experiments. **(B)** Only male mice show a significant reduction in binge-like ethanol intake when the CRF+ CeA-LH circuitry is inhibited. **(C)** BECs show a similar reduction during CNO treatment in male mice only (dotted line indicates 80 mg/dL). **(D)** No sex-specific blunting is seen in female or male mice for sucrose intake. **(E)** Cannula placement for Gi-DREADD animals; placements are marked to the left (filled circles) for female and to the right (open circles) for male animals. Due to the cannula being placed within a pedestal, only 1 hemisphere is shown for placement because each subject was consistent across hemispheres. **(F**–**H)** No significant changes are seen in **(F)** ethanol consumption, **(G)** BECs (dotted line indicates 80 mg/dL), **(H)** or sucrose intake in male and female mice depending on vehicle or CNO treatment. **(I)** Cannula placement for mCherry control animals; placements are marked to the left (filled circles) for female animals and to the right (open circles) for male animals. Data are represented as mean ± SEM. ∗*p* < .05. BEC, blood ethanol concentration; CeA, central nucleus of the amygdala; CNO, clozapine *N*-oxide; CRF, corticotropin-releasing factor; DREADD, designer receptor exclusively activated by designer drugs; LH, lateral hypothalamus.

Figure 1 shows a schematic of virus placement into the CeA and cannulae placement into the LH, as well as photomicrographs of virus expression in the CeA and terminal expression in the LH. Figure 2A shows the timeline of manipulations in the chemogenetic experiment. Planned comparisons for vehicle and clozapine *N*-oxide (CNO) injection groups of each sex showed that CNO injections significantly reduce binge-like ethanol drinking in male (*p* = .009) but not female (*p* = .861) mice (Figure 2B). Similarly, there was a significant reduction in BECs for male mice injected with CNO compared with male mice injected with vehicle (*p* = .021) but not female mice (*p* = .736) (Figure 2C). For DID sucrose drinking, there was no significant impact of treatment (Figure 2D). Figure 1E shows the cannulae placement map. In an *mCherry* control DREADD (designer receptor exclusively activated by designer drugs) study, there were no group differences in ethanol consumption (Figure 2F), BECs (Figure 2G), or sucrose intake (Figure 2H). Figure 2I shows the cannulae placement map. In summary, activation of Gi-DREADD significantly reduced binge-like ethanol intake and BECs in male but not female mice, and CNO was without effect in control virus–treated animals.

### Pharmacological Inhibition of CRF1R in the LH Blunts Binge-Like Ethanol Intake in Male but Not Female Mice, With No Such Effects Seen During Pharmacological Activation of CRF2R

**Figure 3:**
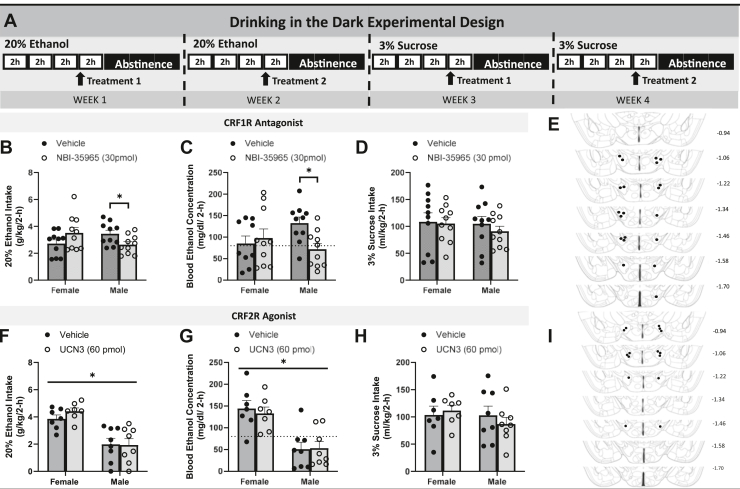
Pharmacological blockade of the CRF1R and stimulation of the CRF2R. **(A)** Timeline of procedures used in the pharmacology experiments. **(B)** Ethanol intake was significantly reduced in male mice treated with NBI-35965, with no such changes in female ethanol consumption. **(C)** BECs of male mice were significantly blunted, while female BEC levels remained the same (dotted line indicates 80 mg/dL). **(D)** No significant changes in sucrose consumption were observed between the sexes. **(E)** Bilateral cannula placements in the LH of all animals. **(F)** No changes in ethanol consumption were observed based on UCN3 or vehicle treatment in either sex. **(G)** Overall, female mice had higher BECs than male mice, but this effect was independent of UCN3 or vehicle treatment (dotted line indicates 80 mg/dL). **(H)** Sucrose drinking was not affected by UCN3 injections in a sex-specific manner. **(I)** Bilateral cannula placements in the LH in all animals. Data are represented as mean ± SEM. ∗*p* < .05. BEC, blood ethanol concentration; CRF1R, CRF_1_ receptor; CRF2R, CRF_2_ receptor; LH, lateral hypothalamus.

To focus on the CRF circuitry within the LH, mice were injected with a CRF1R antagonist (NBI-35965) or CRF2R agonist (UCN3) into the LH. A timeline of procedures from the pharmacology experiments is presented in Figure 3A. Using planned comparisons to assess differences between vehicle and NBI-35965 treatments, there was a significant reduction in ethanol drinking for male mice treated with NBI-35965 (*p* = .03) but not in female mice (*p* = .165) (Figure 3B). Regarding BECs, planned comparisons again showed that the significant CRF1R antagonist–induced reduction in BECs occurred in male (*p* = .0263) but not female (*p* > .9999) mice (Figure 3C). Sucrose drinking was not impacted by pharmacological manipulations (Figure 3D). Figure 3E shows the cannulae placement map for the pharmacology experiment.

Next, involvement of the CRF2R was tested via the use of the selective agonist UCN3. There was no impact of this pharmacological treatment on ethanol intake (Figure 3F), BECs (Figure 3G), or sucrose intake (Figure 3H). Figure 3I shows the cannula placement for the UCN3 experiment. In summary, pharmacological experiments showed that the LH CRF1R signaling modulates binge-like ethanol intake in male but not female mice, which is consistent with the results of the chemogenetic study.

### Repeated Cycles of Binge-Like Ethanol Consumption Alter *Crf* and *Crf* Receptor mRNA in the Amygdala but Not in the LH

**Figure 4:**
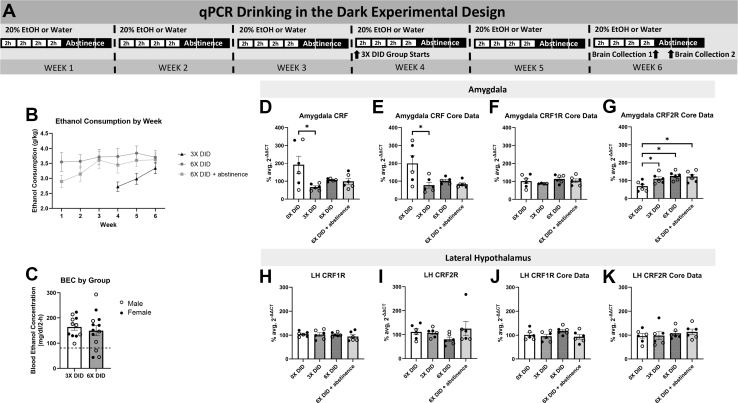
Effects of repeated cycles of binge-like ethanol intake on CRF, CRF1R, and CRF2R mRNA. **(A)** Timeline of procedures used in the qPCR experiments. Control mice received water only throughout the experiment (0× DID), and experimental mice experienced 3 (3× DID) or 6 (6× DID and 6× DID+abstinence) 4-day DID cycles. The 3× DID and 6× DID groups were sacrificed immediately after the last ethanol drinking session, while the 6× DID+abstinence group was sacrificed 24 hours after the last ethanol drinking session. **(B**, **C)** No group differences in **(B)** ethanol consumption or **(C)** BECs (dotted line indicates 80 mg/dL) were observed during the final week of DID. **(D)** CRF mRNA expression in the amygdala was significantly reduced following 3 cycles of DID, **(E)** and the same effect was observed by the UNC Advanced Analytics Core. **(F)** No group differences were seen in CRF1R expression in the amygdala. **(G)** Mice that received repeated cycles of DID with and without a 24-hour period of abstinence demonstrated greater CRF2R mRNA expression in the amygdala than water control mice. **(H**–**K)** No significant group differences were observed for **(H)** CRF1R mRNA expression in the LH or **(I)** CRF2R mRNA expression in the LH, **(J, K)** an effect that was replicated by the UNC Advanced Analytics Core. Data are represented as mean ± SEM. Female data are indicated by filled data points; male data are indicated by open data points. ∗*p* < .05. BEC, blood ethanol concentration; CRF, corticotropin-releasing factor; CRF1R, CRF_1_ receptor; DID, drinking in the dark; EtOH, ethanol; LH, lateral hypothalamus; mRNA, messenger RNA; qPCR, quantitative polymerase chain reaction.

A timeline of experimental procedures from the qPCR experiment is presented in Figure 4A. Ethanol consumption and BECs associated with the mRNA study are shown in Figure 4B and C, respectively. All groups consumed equal amounts of ethanol during the final week of DID, ensuring that differences in mRNA expression were the result of the number of DID cycles received. The effects of binge-like ethanol intake on *Crf* and *Crf* receptor mRNA in the amygdala are presented in Figure 4D and E. Analyses revealed significant group differences, and a planned comparison showed that mice that received 3 cycles of DID had significantly less *Crf* mRNA than water control mice (Figure 4D) (*p* = .042). As seen in Figure 4E, the same effects of binge-like ethanol consumption on *Crf* mRNA in the amygdala were observed from the qPCR data generated by the Advanced Analytics Core. Additionally, data from the Advanced Analytics Core (Figure 4F, G) revealed no effect of binge-like ethanol intake on *Crf1r* mRNA in the amygdala, but there was an effect of binge-like ethanol consumption on *Crf2r* mRNA expression in the amygdala, where planned comparisons revealed that mice that received 3 cycles of DID, 6 cycles of DID, or 6 cycles of DID followed by a 24-hour period of abstinence showed greater *Crf2r* mRNA expression in the amygdala than water control mice (*p* = .049, *p* = .009, and *p* = .014, respectively). The effects of binge-like ethanol consumption on *Crf* receptor mRNA in the LH are shown in Figure 4H–K, and all analyses failed to achieve statistical significance. In summary, a history of binge-like ethanol intake was associated with reduced *Crf* mRNA and increased *Crf2r* mRNA in the amygdala, with no impact on mRNA in the LH.

### CeA Deletion of CRF

**Figure 5:**
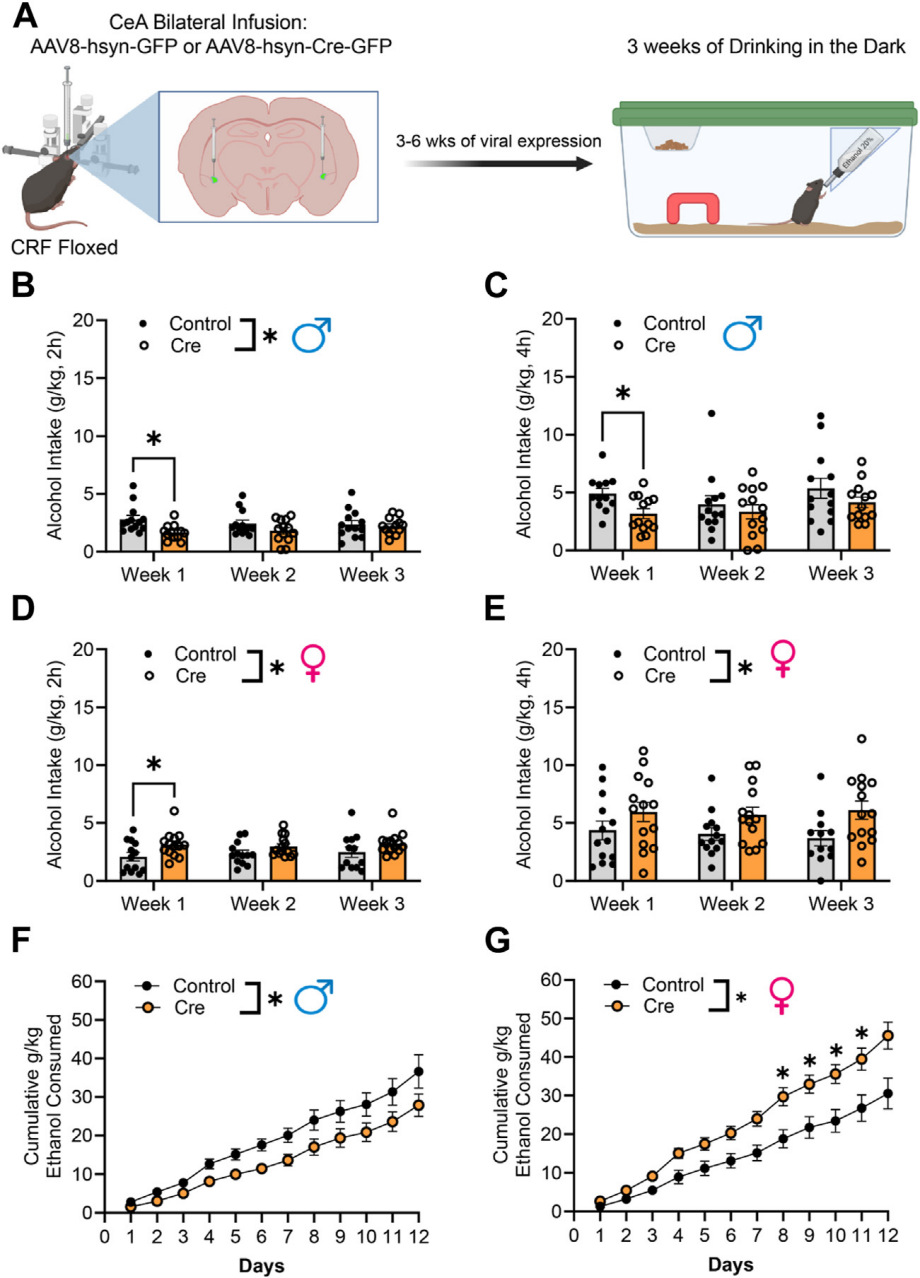
Genetic deletion of CRF in CeA neurons. **(A)** Experimental workflow beginning with bilateral AAV infusion to the CeA of CRF floxed mice followed by 3 to 6 weeks of recuperation before engaging in 3 weeks of DID. **(B)** CeA CRF genetic deletion in CRF floxed male mice decreased average weekly ethanol intake of the 2-hour period in the first week of DID. **(C)** CeA CRF genetic deletion in CRF floxed male mice decreased average weekly ethanol intake of the 4-hour period during the first week of DID. **(D)** CeA CRF genetic deletion increased average weekly ethanol intake of the 2-hour period in the first week of DID of the CRF floxed female mice. **(E)** CeA CRF genetic deletion had an overall increase on the weekly ethanol intake of the 4-hour period in CRF floxed female mice despite each individual week showing no statistically significant differences. **(F)** CeA CRF genetic deletion reduced the cumulative intake of male CRF floxed mice despite no individual day indicating statistically reduced intake. **(G)** CeA CRF genetic deletion increased overall cumulative intake in female CRF floxed mice, with days 7 to 10 showing statistically significant differences compared with control female mice. Closed circles denote AAV8-hsyn-GFP-infused mice (control), while open circles represent AAV8-hsyn-GFP-Cre-infused mice (Cre). Error bars represent SEM. ∗*p* < .05. AAV, adeno-associated virus; CeA, central nucleus of the amygdala; CRF, corticotropin-releasing factor; DID, drinking in the dark.

To determine whether CRF produced in the CeA plays a role in alcohol drinking, we knocked down CRF in the CeA and measured alcohol consumption in male and female mice using the DID paradigm (Figure 5A). Fluorescence in situ hybridization was used to validate *Crh* deletion, and the results of an iunpaired Student’s *t* test (*p* = .0127) suggested that there was a significant decrease in *Crh* punctae in Cre-treated CRF floxed mice compared with control-treated floxed mice, indicating that the *Crh* genetic deletion model worked as intended (Figure S2). Male data are presented in Figure 5B, C, F, and female data are presented in Figure 5D, E, G. Given the known differences in CRF function in the CeA (29), we opted to analyze male and female mice separately. When comparing male control- and Cre-treated mice on weekly 2-hour consumption (Figure 5B), Šídák multiple comparison post hoc tests between male control- and Cre-treated CRF floxed mice indicated a significant decrease in 2-hour weekly average alcohol consumption only at week 1 (*p* = .0167), with no significant difference in week 2 (*p* = .2945) or week 3 (*p* = .9622). When we compared the weekly 4-hour alcohol intake of male control- and Cre-treated CRF floxed mice (Figure 5C), Šídák multiple comparison post hoc tests between male control- and Cre-treated CRF floxed mice indicated a significant decrease in 4-hour weekly average alcohol consumption only at week 1 (*p* = .0290), with no significant difference in week 2 (*p* = .8855) or week 3 (*p* = .5381). Notably, when we compared cumulative daily drinking of male control- and Cre-treated CRF floxed mice (Figure 5F), 2-way repeated-measures analysis of variance suggested that there was a main effect of time (*F*_1.177,25.89_ = 133.8, *p* < .0001), viral treatment (*F*_1,22_ = 4.946, *p* = .0367), and a significant time-by-treatment interaction (*F*_11,242_ = 2.031, *p* = .0263). While there was an overall reduction in drinking in the male Cre-treated CRF floxed mice, Šídák multiple comparisons post hoc tests indicated that this was not statistically significant at any of the 12 days.

When we compared female control- and Cre-treated CRF floxed mice’s 2-hour weekly average alcohol consumption (Figure 5D), in contrast to male mice, Šídák multiple comparisons post hoc tests indicated a significant increase in alcohol intake between control- and Cre-treated female mice in week 1 (*p* = .0328) but not week 2 (*p* = .1085) or week 3 (*p* = .1551). Furthermore, when we compared female control- and Cre-treated CRF floxed mice’s weekly 4-hour alcohol intake (Figure 5E), while a mixed-effects analysis showed that there was a main effect of viral treatment (*F*_1,25_ = 4.471, *p* = .0446), Šídák multiple comparisons post hoc tests indicated no significant differences between control- and Cre-treated female mice in weeks 1 (*p* = .4619), 2 (*p* = .1874), or 3 (*p* = .0772). Notably, when we compared the cumulative daily drinking of female control- and Cre-treated CRF floxed mice (Figure 5G), Šídák multiple comparison post hoc tests indicated that this increase in alcohol intake was statistically different in days 7 (*p* = .0429), 8 (*p* = .0393), 9 (*p* = .0499), and 10 (*p* = .0482). Interestingly, we conducted an exploratory analysis comparing deletion of CRF in male and female mice and found that in all drinking assessments, there was a sex-by-virus interaction, supporting potential differential effects of deletion. Figure S3 illustrates the representative location where maximum virus was localized per animal for the control and Cre CRF floxed male and female mice.

### CeA Deletion of CRF1R

**Figure 6:**
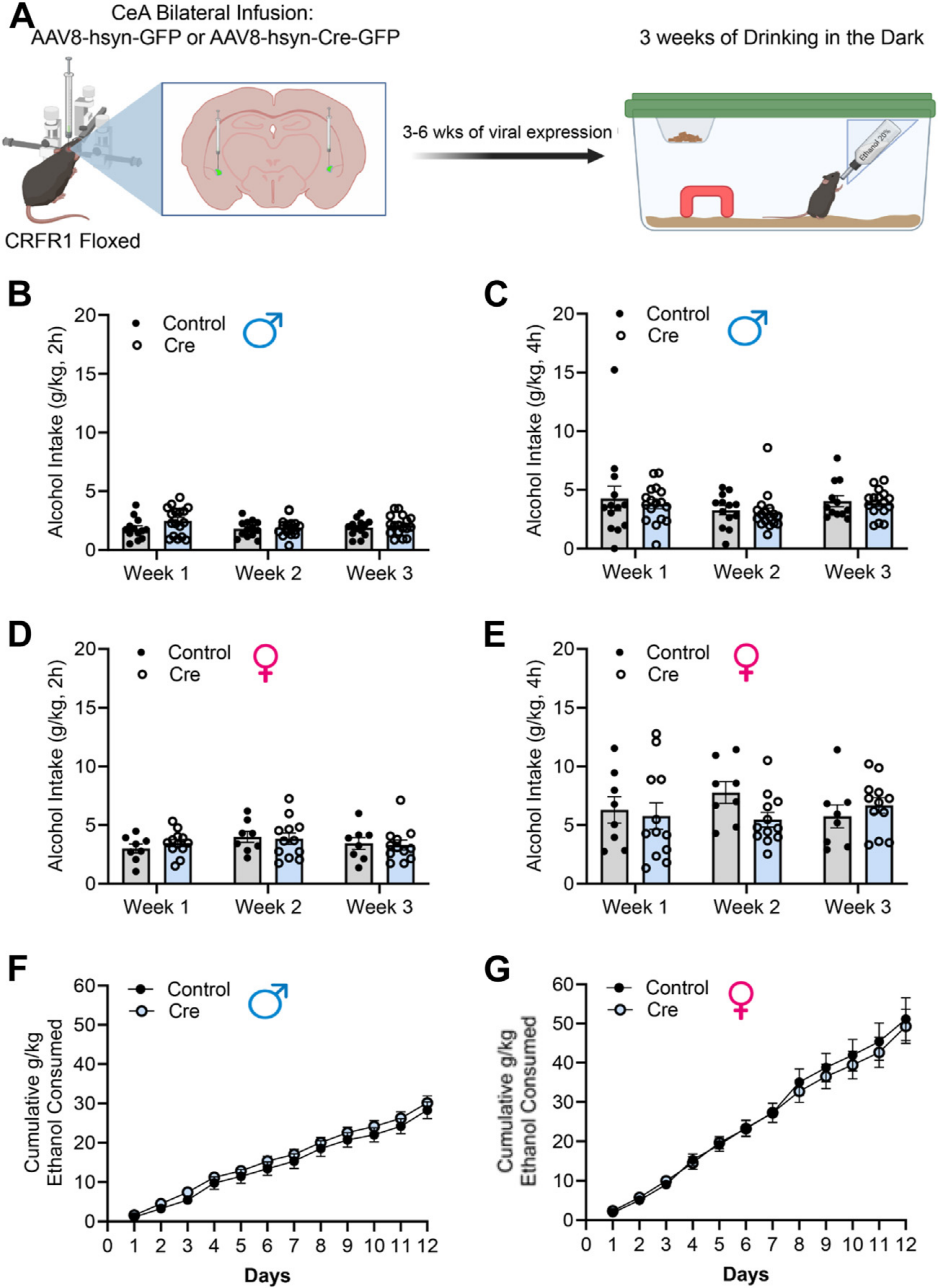
Genetic deletion of CRF1Rs on CeA neurons. **(A)** Experimental workflow beginning with bilateral AAV infusion to the CeA of CRFR1 floxed mice followed by 3 to 6 weeks of recuperation before engaging in 3 weeks of DID. **(B)** No effect of CeA CRFR1 genetic deletion on the average weekly ethanol intake of the 2-hour period in CRFR1 floxed male mice. **(C)** No effect of CeA CRFR1 genetic deletion on the weekly ethanol intake of the 4-hour period in CRFR1 floxed male mice. **(D)** No effect of CeA CRFR1 genetic deletion on the average weekly ethanol intake of the 2-hour period in CRFR1 floxed female mice. **(E)** No effect of CeA CRFR1 genetic deletion in the weekly ethanol intake of the 4-hour period in CRFR1 floxed female mice. **(F)** No effect of CeA CRFR1 genetic deletion on the cumulative intake of male CRFR1 floxed mice. **(G)** No effect of CeA CRFR1 genetic deletion on the cumulative intake of female CRFR1 floxed mice. Closed circles denote AAV8-hsyn-GFP infused mice (control), while open circles represent AAV8-hsyn-GFP-Cre-infused mice (Cre). Error bars represent SEM. AAV, adeno-associated virus; CeA, central nucleus of the amygdala; CRF, corticotropin-releasing factor; DID, drinking in the dark.

We wanted to determine whether local CeA CRF1R plays a role in binge-like alcohol drinking (Figure 6A). Fluorescence in situ hybridization was used to validate *Crhr1* deletion, and an unpaired Student’s *t* test (*p* = .0166) indicated that there was a significant decrease in *Crhr1* punctae in Cre-treated CRF1R floxed mice compared with control-treated CRF1R floxed mice (Figure S4). This indicates that the *Crhr1* genetic deletion model works as intended. Male data are presented in Figure 6B, C, F, and female data are presented in Figure 6D, E, G. When we compared both male and female control- and Cre-treated CRF1R floxed mice, there was no main effect of viral treatment or time, and no time-by-viral treatment interaction for 2-hour weekly average alcohol consumption, 4-hour weekly alcohol consumption, or cumulative alcohol consumption (Figure 6B–G). This suggests that CRF1R synthesized in CeA neurons does not play a critical role in binge-like alcohol consumption. Figure S5 illustrates the representative location where maximum virus was localized per animal for the control and Cre CRF1R floxed male and female mice. In summary, genetic deletion of CRF from the CeA was associated with reduced ethanol intake in male mice but increased ethanol intake in female mice, while deletion of CRF1R from the CeA was without effect.

## Discussion

Our results here show that chemogenetically silencing a CRF+ circuit between the CeA and LH, CRF1R blockade in the LH, and deletion of CRF from the CeA were treatments that were more sensitive in blunting binge-like ethanol drinking and BECs in male mice. It is not surprising that these manipulations did not completely prevent binge-like ethanol intake in male mice because this is only one of numerous circuits that have been shown to modulate binge-like ethanol intake (30, 31, 32, 33, 34, 35, 36). Nonspecific (21) and CRF1R+ (22) CeA-LH CRF+ neurocircuitry have been mapped previously in rats and associated with avoidance of stress-associated stimuli and stress-induced anxiety-like behavior and ethanol self-administration, and we provide evidence of a CRF+ CeA→LH circuit in our CRH-ires-cre mice here. We extend the findings of Weera *et al.* by showing that CRF+ CeA→LH neurocircuitry modulates binge-like ethanol intake in male mice only under the current experimental conditions. Furthermore, the CeA→LH neurocircuitry that we identified here acts as a specific neuronal target in binge-like ethanol consumption because chemogenetic silencing of this pathway had no effect on sucrose consumption in either sex. In addition, these effects were not a byproduct of CNO manipulation; by itself, CNO failed to cause any significant changes in ethanol consumption.

Pharmacological inhibition of CRF1R in the LH reciprocated chemogenetic findings by having greater sensitivity in blunting DID ethanol intake and associated BECs in male mice. Activation of the CRF2R did not significantly alter binge-like ethanol intake in male or female mice. Previous work in other brain regions has found that pharmacological inhibition of CRF1R and activation of CRF2R lead to a reduction in ethanol consumption (34,37); however, our current results suggest that in the LH, at the doses tested, only the CRF1R is involved in modulating ethanol intake and only in male mice. Given evidence of sex differences in amygdala CRF1 binding (38), testing with higher doses of the CRF1R antagonist in the LH is necessary to confirm a lack of involvement in female ethanol intake or whether the CRF1R is involved but is less sensitive (requires higher doses) in female mice. Similarly, testing with higher doses of the CRF2R agonist is necessary before a role of this receptor can be firmly ruled out. Consistent with chemogenetic and pharmacological data, we found that genetic deletion of CRF from CeA neurons significantly blunted cumulative ethanol intake in male mice but increased it in female mice, while genetic deletion of CRF1R from the CeA did not alter ethanol intake in either sex. Finally, we found that a history of binge-like ethanol intake was associated with decreased *Crf* mRNA and elevated *Crf2r* mRNA in the CeA regardless of sex.

The sexually dimorphic role of CRF signaling, particularly via the CRF1R, has been observed previously. Therefore, compared with males, female *Crf* mRNA, CRF1R binding, and the number of CRF+ neurons are upregulated through a number of brain regions such as the paraventricular nucleus of the hypothalamus (28,39, 40, 41), amygdala (28,38), and bed nucleus of the stria terminalis (42,43). At the level of cell signaling, acute application of ethanol onto mouse CeA slice preparations reduced GABA release onto CRF1R-expression neurons in male but not female mice, and application of exogenous CRF increased the firing rate of CRF1R-expressing neurons to a greater extent in male mice (29). Taken together, sex differences in CRF system organization and sensitivity may account for the observed sex differences in chemogenetic, genetic deletion, and pharmacology manipulations in the current report.

As with our pharmacological manipulations, using higher doses of CNO may reveal a role for the CRF+ CeA→LH circuitry in modulating ethanol intake in female mice, which would support the hypothesis of sex differences in the sensitivity of this CRF circuit. Previously, female mice expressing Gi-DREADD in CRF+ bed nucleus of the stria terminalis neurons required a higher dose of CNO to blunt ethanol binge drinking than male mice, suggesting that female mice are less sensitive to the effects of CNO on DREADD receptors (44). Furthermore, intra–medial prefrontal cortex administration of CNO to activate Gi-DREADD on NPY1R+ basolateral amygdala terminals dose-dependently decreased ethanol intake in male and female animals such that a low dose of CNO was effective at blunting ethanol consumption in male mice, while a high dose of CNO was required to achieve the same results in female mice (35). In the current experiment, we did not assess different dosages of CNO. Thus, in light of the evidence noted above regarding reduced CNO sensitivity in female mice, it is possible that our current findings of a male-specific CRF+ CeA→LH circuit in the modulation of binge-like ethanol intake may be recapitulated in female mice when using a higher dose of CNO.

It is unlikely that LH CRF1R signaling modulates binge-like ethanol intake via a mechanism that involves hypothalamic-pituitary-adrenal axis functioning because binge-like ethanol intake has not been found to alter hypothalamic-pituitary-adrenal axis activity (18). Consistently, repeated cycles of binge-like ethanol intake did not alter CRF immunoreactivity in the paraventricular nucleus of the hypothalamus but did increase CRF immunoreactivity in the CeA, and this change lasted 18 to 24 hours into abstinence in male mice (13). These previous observations and the current data suggest that while hypothalamic CRF1R signaling modulates binge-like ethanol intake, this signaling likely stems from extrahypothalamic sources of CRF (i.e., from the CeA) and does not involve hypothalamic pools of CRF that are involved with hypothalamic-pituitary-adrenal axis function.

We also found that repeated cycles of binge-like ethanol consumption altered *Crf* and *Crf2r* mRNA expression in the amygdala but not amygdala *Crf1r* mRNA expression. Mice that received 3 cycles of DID showed significantly reduced *Crf* mRNA expression in the amygdala compared with water-drinking control mice, while mice that received 3 or 6 cycles of DID showed significantly greater *Crf2r* mRNA expression in the amygdala compared with water-drinking control mice. *Crf2r* expression remained significantly elevated 24 hours into abstinence. Previous research has demonstrated that CRF protein expression in the amygdala is elevated following binge-like ethanol consumption (13), and *Crf* mRNA expression has been found to increase following ethanol dependence in rats (45). We hypothesize that our data provide evidence for a compensatory mechanism whereby a decrease in *Crf* mRNA in the amygdala following binge-like ethanol consumption may serve as an attempt to attenuate increased CRF signaling and protein levels following ethanol intake, and failure to regulate this CRF system may contribute to the development of ethanol dependence, supporting an allostatic view of neurobiological changes in response to ethanol (46). Neither *Crf1r* nor *Crf2r* mRNA expression in the LH was impacted by binge-like ethanol consumption. One limitation of the current dataset is that due to the small size of these brain regions in mice, 2 tissue samples were pooled to obtain sufficient mRNA for complementary DNA synthesis, effectively halving our sample size. Therefore, it is likely that the current qPCR study was underpowered to detect sex differences in mRNA expression. However, an inspection of the individual data points from the mRNA data (Figure 4) did not suggest any obvious trends for sex differences in expression patterns despite sex differences in ethanol intake.

One of the key questions that can arise through the use of DREADD approaches is: What is the cellular effector driving the change in behavior? For our work, one important question was whether CRF from the CeA drove these behavioral changes. While our pharmacology studies provided strong support for this idea, the genetic approach with site-specific deletion could provide converging evidence. Consistent with the pharmacology, we found that deletion of CRF in the CeA reduced ethanol consumption in male mice but increased consumption in female mice. These effects were most pronounced during the first week of DID testing, which may reflect some form of time-dependent compensatory neuroplasticity or a more key role for this system early in our experiment, although cumulative treatment effects were observed over all days of DID testing. The increase in ethanol drinking in female mice is especially intriguing when considering our DREADD findings because it suggests an important sex difference in the modulatory systems and circuits that regulate behavior. Because there is evidence that CRF1R can act as a presynaptic heteroreceptor to regulate CRF and GABA release (34,45,47) and because CRF neurons in the CeA of CRH-ires-cre mice show GABAergic and CRF1R phenotypes (48), we deleted CRF1R from neurons of the CeA under the hypothesis that this would blunt CRF1R trafficking to axons of projection sites, including in the LH. The deletion of CRF1R in the CeA had no effect on ethanol consumption. This is in contrast to the pharmacological studies supporting a role for CRF1R in the CeA in alcohol drinking (13). These results suggests that presynaptic CRF1Rs on CRF terminals in the LH from neurons arising in the CeA are likely not involved, and thus, it will be important in future studies to delete CRF1R within the LH to ascertain the potential role of postsynaptic CRF1Rs in the LH. It is also possible that the level of deletion that we found was insufficient to drive changes in function. Another limitation of our study is that virus spread leaked into other neighboring regions of the CeA including the basolateral amygdala, which means that we cannot rule out possible unwanted deletion of CRF or CRF1R in those regions causing nonspecific effects.

### Conclusions

The current observations reveal a novel CRF+ circuit between the CeA and LH that is more sensitive in modulating binge-like ethanol intake in male mice than female mice. Given the abundance of research demonstrating that female rodents consume significantly more ethanol than male rodents, the current findings may represent a critical circuit that contributes to sex differences in ethanol intake. Theoretically, blunted sensitivity of this circuit in female mice may contribute to their elevated levels of ethanol intake. The current observations may also provide insight into sex-dependent treatment approaches for AUD.

## Supplementary Material

Document S1 Supplemental Methods, Supplemental Results, and Figures S1-S6

Key Resources Table
