# Supplementary material for: Corticotropin-Releasing Factor Modulates Binge-Like Ethanol Drinking in a Sex-Dependent Manner: Impact of Amygdala Deletion and Inhibition of a Central Amygdala to Lateral Hypothalamus Circuit
Source: Biol Psychiatry Glob Open Sci. 2024 Oct 25;5(1):100405. doi: 10.1016/j.bpsgos.2024.100405 (PMC11629220; doi:10.1016/j.bpsgos.2024.100405)
Supplement: Document S1 — Supplemental Methods, Supplemental Results, and Figures S1-S6 [file mmc1.pdf]

SUPPLEMENTARY INFORMATION

Corticotropin-Releasing Factor Modulates Binge-Like Ethanol Drinking in a Sex-Dependent Manner: Impact of Amygdala Deletion and Inhibition of a Central Amygdala to Lateral Hypothalamus Circuit

Bendrath *et al.*

Supplemental Figures

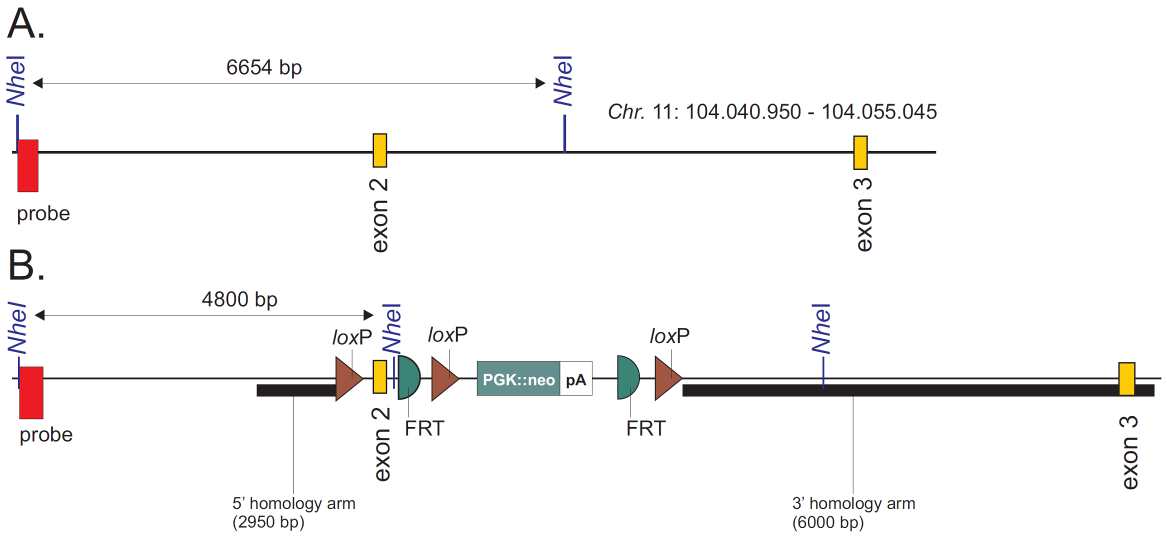

Fig. S1: Targeting construct for generation of floxed CRFR1 mice

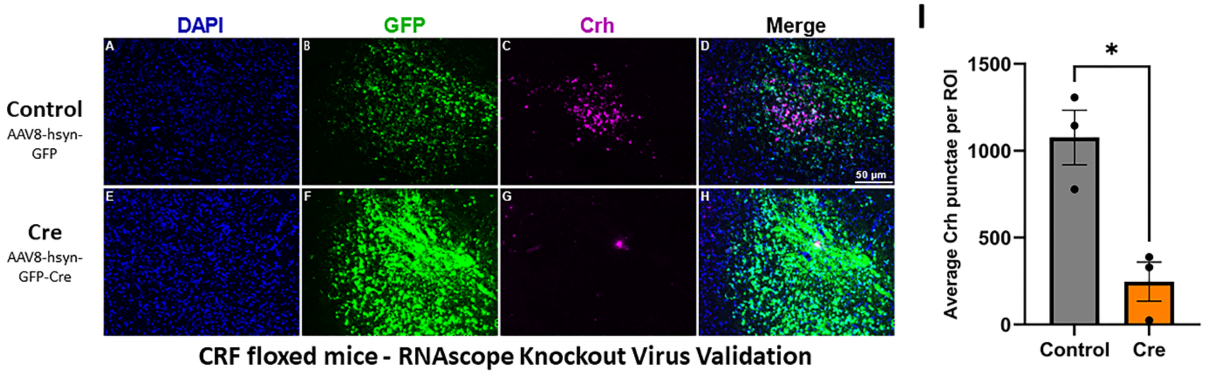

Fig. S2: Validation of CRF genetic deletion. (A-D) Representative image showing Crh expression in the CeA of control treated mice (E-H) Representative image showing Crh expression in the CeA of Cre treated mice (I) Significant reduction of average Crh punctae per ROI in Cre treated mice. AAV8-hsyn-GFP treated CRF floxed mice (Control), AAV8-hsyn-GFP-Cre treated CRF floxed mice (Cre), scalebar 50um, each data point represents average punctae per ROI from each mouse, Control treated group had, N=3 mice, 4-10 serial sections per mouse, while Cre treated group had, N=3 mice, 2-5 serial sections per mouse. Crh was only counted in ROI's that had GFP expression indicative of virus presence. $^{*}p < 0.05$. DAPI, denotes nucleus of cells in blue, GFP denotes viral GFP tag in green, Crh in magenta denotes mRNA punctae of Crh. Error bars are represented as SEM, $^{*}p < 0.05$.

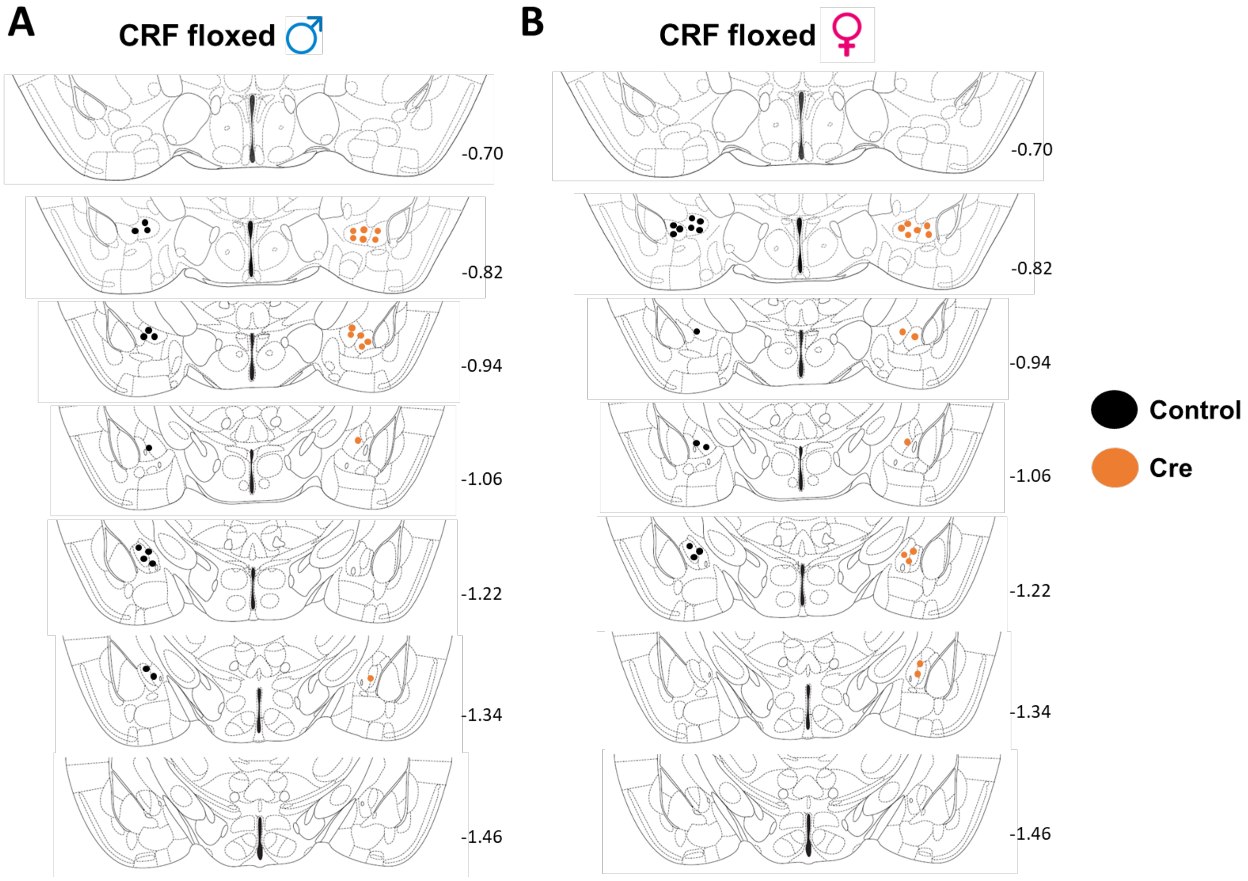

Fig. S3: (A) Representative location relative to bregma where maximum virus was localized in male CRF floxed mice. (B) Representative location relative to bregma where maximum virus was localized in female CRF floxed mice. 1 dot per mouse, black dots = Control mice and orange dots = Cre mice.

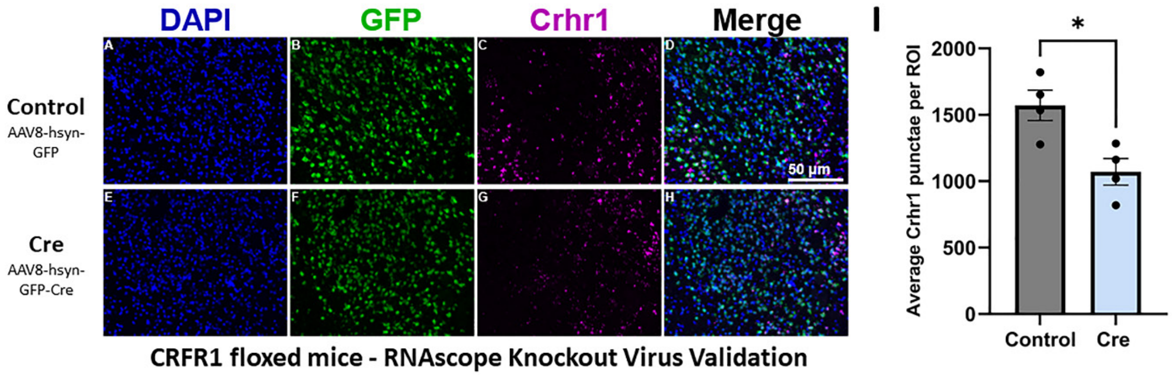

Fig. S4: Validation of CRF1R genetic deletion. (A-D) Representative image showing Crhr1 expression in the CeA of control treated CRFR1 floxed mice (E-H) Representative image showing Crhr1 expression in the CeA of Cre treated CRFR1 floxed mice (I) Significant reduction of average Crhr1 punctae per ROI in Cre treated CRFR1 floxed mice. AAV8-hsyn-GFP treated CRFR1 floxed mice (Control), AAV8-hsyn-GFP-Cre treated CRFR1 floxed mice (Cre), scalebar 50um, each data point represents average punctae per ROI in each mouse, N=4 mice, 5-9 serial sections per mouse, while Cre treated group had, N=4 mice, 4-9 serial sections per mouse. Crhr1 was only counted in ROI's that had GFP expression indicative of virus presence. Crhr1 was only counted in ROI's that had GFP expression indicative of virus presence. *$p < 0.05$. DAPI, denotes nucleus of cells in blue, GFP denotes viral GFP tag in green, Crhr1 in pink denotes mRNA punctae of Crh. Error bars are represented as SEM

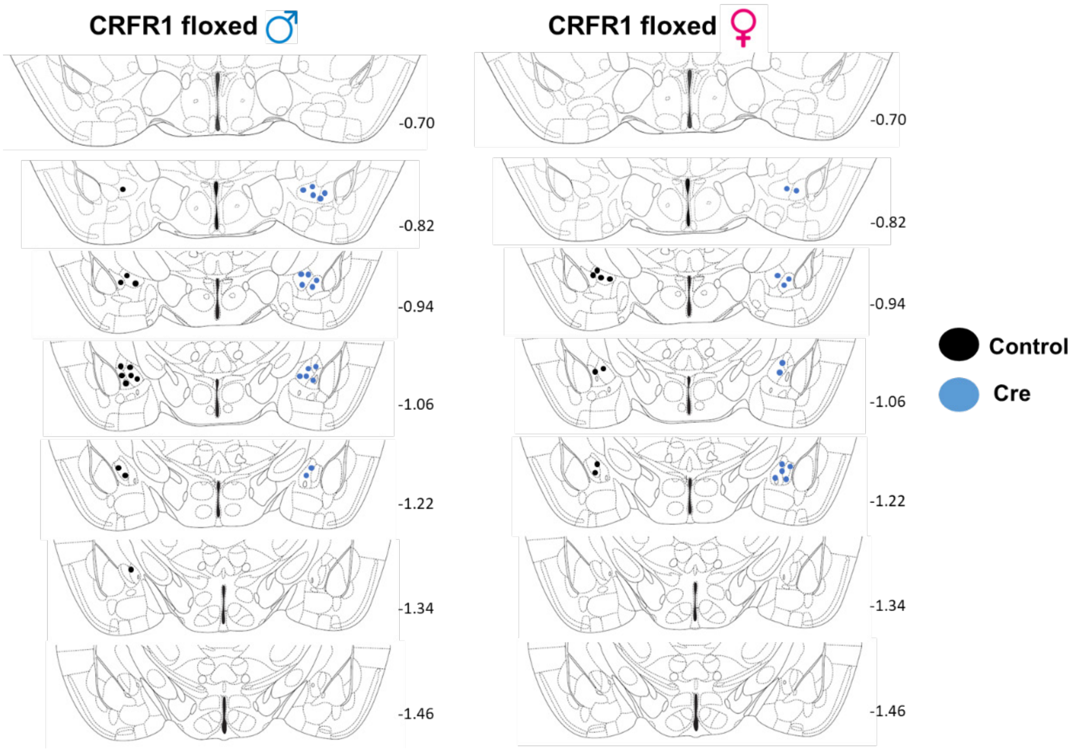

Fig. S5: (A) Representative location relative to bregma where maximum virus was localized in male CRFR1 floxed mice. (B) Representative location relative to bregma where maximum virus was localized in female CRFR1 floxed mice. 1 dot per mouse, black dots = Control mice and blue dots = Cre mice.

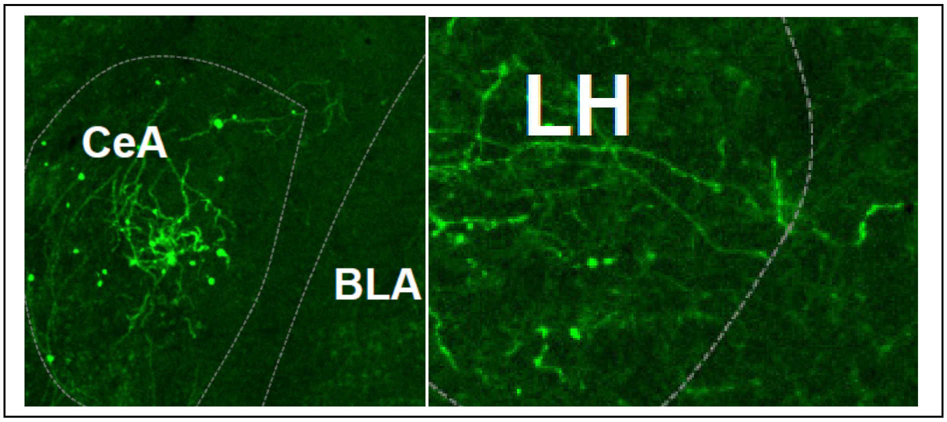

Fig. S6: CRH-ires-cre mice were given of a cre-inducible reporter virus (AAV5-EF1a-DIO-hChR2-mCherry; 0.3 µl bilaterally) into the central amygdala (CeA) and 3-weeks later slices were collected containing the CeA (and basolateral amygdala; BLA) as well as the lateral hypothalamus (LH). Soma of CRF+ neurons are evident in the CeA (left panel) and CRF+ terminals a are evident in the LH (right panel).

METHODS

Animals

Male and female C57BL/6J mice (Jackson Laboratories, Bar Harbor, ME) aged 8-10 weeks at the start of experimentation were used for the pharmacological study (CRF1-antagonist: male, n=20; female n=20) and the qPCR study (male, n=25; female, n=25). The *in vivo* chemogenetic experiments used 29 male and female (Gi-DREADD male, n=7; Gi-DREADD female, n=8; control virus male, n=8; control virus female, n=6) CRH-ires-Cre (CRH-Cre) mice (positive for the expression of Cre under the CRH promoter as determined by standard PCR genotyping protocols) at least 10 weeks of age. CRH-Cre mice were generated and genotyped as described previously (1). For the Crh deletion

experiment, 27 CRF floxed males (Control treated male, n=14; Cre treated male, n=13) and 27 CRF floxed females (Control treated female, n=13; Cre treated female, n=14) aged 11-13 weeks at the start of behavioral testing were used with mice receiving either infusion of an AAV8-hsyn-gfp (Control) or AAV8-hsyn-cre-gfp (Cre) into the CeA as described in the methods section. Floxed CRF mice were generated as described previously (2). For the Crhr1 deletion experiment, 31 CRFR1 floxed male (Control treated male, n=14; Cre treated male, n=17) and 20 CRF1R floxed females (Control treated female, n=8; Cre treated female, n=12) aged 15-22 weeks at the start of behavioral testing were used with mice receiving either infusion of an AAV8-hsyn-cre-gfp (Cre) or a AAV8-hsyn-GFP (Control) into the CeA as described below. CRF1R floxed mice were generated as described in the supplementary information extended methods. All mice were housed individually in an AAALAC accredited vivarium on a reverse 12 h light-dark cycle, with the lights going off at 09:30 or 10:30 hours, depending on the room. Water (unless otherwise stated) and food (Prolab® RMH 3000 (Purina LabDiet®; St. Louis, MO)) were available to all animals ad libitum. All procedures were approved by the University of North Carolina Institutional Animal Care and Use Committee, and conducted in accordance with the Guidelines for the Care and Use of Laboratory Animals.

<u>*DREADD and Cannula Surgery*</u>

Surgeries were conducted on an Angle II™ Stereotax (Leica Instruments, Buffalo Grove, IL). Animals received intraperitoneal (i.p.) injections (1.5 mL/kg) of ketamine (117 mg/kg)/ xylazine (7.92 mg/kg), with all coordinates being measured from bregma. For the *in vivo* chemogenetic studies, CRH-ires-cre mice from the same colony received either an injection of Cre-dependent control vector (AAV8-hSyn-DIO-mCherry, Addgene,

Watertown, MA) or the Cre-dependent Gi/o-coupled DREADD vector (AAV8-hSyn-DIO-hM4d-mCherry, Addgene, Watertown, MA) into the CeA (AP -1.06, ML +/- 2.42, DV -4.63). The control virus study was run to demonstrate that clozapine-N-oxide (CNO), relative to vehicle infusion, would not have independent effects on behavior in the absence of the Gi-DREADD, a concern that has been raised elsewhere (3). The injection needle remained in place for 10-15 additional minutes before being withdrawn. This Designer Receptor Exclusive Activated by Designer Drugs (DREADD) has been validated and used to manipulate CRF activity in the central amygdala previously (4-6). Additionally, bilateral guide cannulas (Plastics One; Roanoke, VA) were implanted into the LH (AP -1.10, ML +/- 1.10, DV -5.10; injector: 5MM with 0.5MM projection). Upon surgery completion, all mice recovered for at least 3 weeks before starting behavioral testing for maximal viral expression. For the *in vivo* pharmacological study, animals were implanted with bilateral guide cannulas in the same LH coordinates for intra-LH microinfusions, and were allowed to recover from surgery for 1 week before starting the DID procedure.

Genetic Deletion Surgery

Surgeries were conducted on Kopf Stereotaxic device (Kopf Instruments, Tujunga, CA, USA). Mice were first anesthetized by placing inside a chamber that contained vaporized 4% isoflurane and oxygen and then maintained via at 2-3% isoflurane during surgery. All coordinates being measured from bregma. A Hamilton syringe infused 250nl of either control virus (AAV8-hsyn-GFP) and titer of 4.1X10e12 or Cre virus (AAV8-hsyn-Cre-GFP) titer of 4.5X10e12 to the CeA at (-0.62, ML +/- 2.70, DV -5.00 or -0.80, ML +/- 2.95, DV -4.85). The injection needle remained in place for approximately 7 additional minutes per injection site before being withdrawn. Upon surgery completion, all mice recovered for at

least 3 to 6 weeks before starting behavioral testing for maximal viral expression. For the study we included mice with CeA bilateral and unilateral hits, a hit was defined as having viral tag GFP presence in the CeA.

"Drinking in the Dark" (DID) Procedures

A 4-day DID model was used to assess ethanol intake, which is a standard protocol to induce binge-like ethanol consumption (7). For the chemogenetic, pharmacology, and PCR studies mice had 2-hours of ethanol access on each of the 4 DID days. For the genetic deletion studies, mice had access to ethanol for 2-hour on days 1-3 of the DID procedure and for 4-hours on day 4 of the DID procedure. This procedure involves replacing the cage water bottle with a 20% (v/v) ethanol solution or a 3% (w/v) sucrose solution 3-hours into the animals' dark cycle. Drinking access each day was limited to 2 hours, with ethanol consumption recorded at the end of each access session. The amount consumed by each subject was recorded during hour 1 and hour 2 on the test (4th) day. To correct for spillage, in parallel to each experiment we ran a drip bottle, 20% ethanol bottle in an empty cage. Sucrose drinking control animals went through the same DID procedure with a 3% (w/v) sucrose solution, and consumption was recorded at the end of each access session (with 1- and 2-hour consumption levels recorded on the last day). Animals received a 3-day period of abstinence before the new 4-day cycle of DID began. For the genetic deletion experiments, alcohol drinking session consisted of 2 hours during the first three days (Monday-Wednesday) and to 4hrs (Thursday) during the 4th day of each week for 3 weeks, alcohol bottles were weighted after each drinking session each day. Mouse weights were measured at least once a week for the duration of the experiment. There were instances where a mouse weight was accidentally not recorded,

in which case, the estimated weight using the calculated average weight-gain was used. Additionally, there were instances where the drip bottles leaked excessively. In these circumstances, the average drip value of the two other weeks was used. For the qPCR experiment, mice were randomly assigned to one of four groups while ensuring equal distribution of sex and initial body weight. Group one served as the water control group and remained ethanol-naïve for the duration of the experiment. Group two and three received three and six, 4-day cycles of DID, respectively, and brains were extracted immediately following the final session of DID. Group four received six cycles of DID followed by a 24 hour period of abstinence from ethanol, therefore brains were extracted 24 hours following the final session of DID.

Drug Administration

Approximately 30 minutes prior to ethanol access on the 4th day of DID, animals received microinjections with either the DREADD ligand CNO (900pmol, as dose we have successfully used with site-directed infusions (8-10)) or vehicle (1% DMSO in 0.9% saline), in a counterbalanced 2x2 Latin-square design during consecutive weekly DID session. All animals were assigned randomly to either drug or vehicle treatment on the first test day, then received the other treatment on test day 2 during the second DID cycle. Drug injections were performed with a Hamilton syringe (Reno, NV) on a Harvard Apparatus PHD 2000 infusion pump (Holliston, MS) at a rate of 0.10μl for 3 minutes (0.30μl total). After injection, infusion needles stayed in the cannula for an additional minute to ensure complete diffusion. Tail blood samples were collected by nicking the lateral tail vein on the 4th day immediately after ethanol access (about 30μl), to assess

blood ethanol concentrations (BECs) on an (AM1) Alcohol Analyzer (Analox, London, UK).

In Vivo Pharmacology

For CRF1R antagonism, NBI-35965 hydrochloride (Tocris, Bristol, UK: 30pmol/0.3 µl/side) was dissolved in sterile H_2O for a drug make-up of 13µg/ml. Bilateral microinjections of NBI or vehicle occurred about 30 minutes before test day ethanol access (DID day 4), and were administered at a rate of 0.10µl/min to reach the target volume of 0.30µl per side using Hamilton infusion pumps. For CRF2R agonism, Urocortin 3 (Ucn3; GenScript USA, Piscataway, NJ: 60pmol/0.4µl/side) was dissolved in DMSO (10% v/v final concentration; Sigma-Aldrich, St. Louis, MO) and diluted with 0.9% saline, for a target concentration of 250µg/mL. Ucn3 or its vehicle was also administered at a rate of 0.1µl/min 30 minutes before ethanol access. Injectors were left in place for one minute post-infusion to allow for diffusion of the drug away from the injector tip and minimize back flow of drug as the injectors were removed. Again, all animals were assigned randomly to either the drug or vehicle groups, and received the opposite treatment on test days in a 2x2 Latin square design during consecutive weekly DID session.

Generation of Floxed CRFR1 Mice

A phage DNA library was screened in two rounds with a 32P-dCTP-labeled probe corresponding to exon 2 (88 bp) of the Crhr1 gene (MGI: 88498) and identify mouse genomic DNA clones that contains exon 2. Several clones were picked and mapped with a panel of restriction enzymes. Clone 12 was selected and used to create the targeting construct. The construct included 1 kb genomic sequence as 5' arm of recombination, a

first loxP site, ~750 bp genomic sequence containing exon 2, a pair of neighboring frt and loxP sites, a pGK1-neo-pA resistance gene cassette, a second pair of frt and loxP sites, 6 kb genomic sequence as 3' arm of recombination (see Figure), and the herpes simplex virus thymidine kinase (TK) for negative selection. TL-1 embryonic stem (ES) cells derived from 129/SvEvTac mice cells were electroporated with the linearized vector and grown on fibroblast feeder cells in DMEM supplemented with 15% fetal bovine serum, 50 mg/ml gentamicin, 1000 U/ml leukemia inhibitory factor, 90 mM b-mercaptoethanol, 0.2 mg/ml G418 (positive selection), and 2 mM ganciclovir (negative selection). Three hundred and seventy-one independent neomycin-resistant colonies were selected and grown in 96-well plates on feeder layer, expanded, and analyzed for the presence of the mutant gene by performing Southern blot analysis using genomic DNA digested with NheI and hybridized with a 32P-labeled probe consisting of 304 bp upstream of the 5' arm of recombination. Five positive clones (clones showing a 4.8 kB NheI fragment) were selected and screened at the 3' end. Clones 3C6 and 4C4 were confirmed and injected into C57BL/6J blastocysts generating 4 and 3 chimeric animals (>60% brown fur), respectively. After demonstrating germline transmission for line 4C4, the mice were crossed to E2a-CRE mice to obtain partial recombination, i.e. 3 loxP to 2 loxP with the loss of the neomycin-resistance gene cassette. The mice were then bred for additional generations into C57BL6/J prior to study. The targeting construct for generation of floxed CRFR1 mice is presented in **supplemental Fig S1**.

<u>*Fluorescence In Situ Hybridization (FISH):*</u>

In order to confirm genetic deletion of CRF and CRF1R, brain collection, cryosectioning and FISH was performed as previously described (11). Mouse brains were flash frozen

in 2-methylbutane and dry ice and then stored in -80°C. 14μm-thick serial sections were collected using a Leica CM 3050S cryostat (Leica Biosystems), these sections were mounted on Super Frost Plus Slides (Fisher Scientific), 4-5 sections per slide. FISH was performed following the manufacture's protocol for the Advanced Cell Diagnostics RNAscope Fluorescence Multiplex Assay (Advanced Cell Diagnostics). We used the following probes for detecting mRNA puncta, Crh-C1 (Mm-Crh), Crhr1-C2 (Mm-Crhr1-C2), eGFP-C3 (EGFP-O4-C3). In order to fluorescently view the mRNA puncta we used TSA vivid Dyes, opal 570 or opal 650. The nucleus of the cells were counterstained with RNAscope DAPI for visualization. Finally, cover slips were mounted on the slides using ProLong Diamond Antifade Mountant (Thermo Fisher Scientific). Slides were imaged using a VS200 Slide Scanner microscope. Images were analyzed using QuPath software version 0.5.0, and an ROI was created over the CeA. In the ROI, the cell detection function (using a value of a 100-intensity parameter threshold) was used to detect all DAPI-positive cells while the subcellular spot detection function (using a detection threshold of 250), was used to detect Crh or Crhr1 puncta, in DAPI-positive cells. Only ROIs that had GFP, indicating the presence of virus, were included for downstream analysis in this study.

Perfusion and Histology

For cannula placement checks and DREADD expression, mice were prepared for perfusion by administering a 0.1mL/kg i.p. injection of ketamine/xylazine (6.67 mg/0.1 mL; 0.67 mg/0.1 mL; in 0.9% saline). Then, mice were perfused transcardially with 0.1M phosphate buffer saline (PBS; pH=7.4) and10% buffered formalin phosphate (fisher chemical). After extraction, brains were fixed in 10% buffered formalin phosphate for 24-

48 hours, before being sliced at 40μm thickness on a vibratome (Leica VT1000S vibratome; Wetzlar, Germany). For the chemogenetic study, cannula placement and DREADD expression were verified on an optical wide field microscope (Leica DM6000) with a digital camera (Roper Scientific). Cannula placement checks for the pharmacological experiments were conducted using a Nikon e400 biological microscope with a digital sight ds-u1 imaging attachment (Nikon Instruments Inc., Melville, NY, USA). Cannula targets were determined by the end of the guide cannula plus 2mm to account for the injector tip. For the genetic deletion experiments, to assess targeting of AAV in floxed mouse lines, tissue was prepared as before (Bloodgood et al., 2021). Mice were sacrificed one day after completing 3 weeks of DID. Mice were perfused using PBS and 4% PFA. Brains were isolated and stored in 4% PFA and later washed and stored in PBS at 4°C. Brain slices were serially sectioned at 45μm-thick using a vibratome (VT 1200s, Leica Biosystems), and sections were collected in Super Frost Slides and mounted using Vectashield Hardset Antifade mounting media with DAPI (Vector Laboratories). To assess targeting of virus, brain sections were then imaged using a VS200 Slide Scanner (Olympus) with Orca Fusion camera (Hamamatsu). Mice that had bilateral and unilateral hits were included in the genetic deletion study.

Reverse Transcription Quantitative Polymerase Chain Reaction (RT-qPCR)

RNA extraction, cDNA synthesis, and RT-qPCR were performed as previously described (12, 13). For mRNA expression analysis, mice were euthanized via rapid decapitation and trunk blood samples were collected for BEC analysis. Brain tissue was extracted and flash frozen in O.C.T. compound (ThermoFisher Scientific, Waltham, MA). Brains were sliced at 40μm thickness on a cryostat (CM3050 S, Leica, Buffalo Grove, IL) and 1mm

tissue punches were collected bilaterally for the amygdala and LH. Tissue samples from two animals within the same group and sex were pooled for each sample to obtain sufficient RNA for analysis. Tissue punches were collected in bead homogenizing tubes containing 500 mL of TriReagent (Molecular Research Center, Cincinnati, OH). RNA extraction, cDNA synthesis, and RT-qPCR were performed as previously described (Barkell et al., 2022; Paniccia et al., 2018). Briefly, RNA from each sample was extracted and purified using the Qiagen RNEasy Tissue Mini Kit for RNA purification (Qiagen, Hilden, Germany). RNA spectroscopy using the Take3 Application and Gen5 Software for Nucleic Acid Quantification (BioTek Instruments Inc., Winooski, VT) was used to assess the concentration and purity of the RNA in each sample. Each sample was diluted with PCR grade water so that all samples contained an equal concentration of RNA. cDNA was synthesized using the Advantage for RT-PCR Kit (Clontech, Takara, Mountain View, CA) and a Veriti 96 Well Fast Thermal Cycler (Applied Biosystems, ThermoFisher Scientific, Waltham, MA). qPCR was performed using the TaqMan Fast Advanced Master Mix Kit (Applied Biosystems, ThermoFisher Scientific, Waltham, MA) and TaqMan Gene Expression Assays for CRF (Mm04206019_m1), CRF1R (Mm00432670_m1), CRF2R (Mm00438308_m1), and the housekeeping gene BetaActin (Mm01205647_g1). Samples underwent repeated cycles of amplification and data collection using the QuantStudio 6 Flex real-time PCR system (ThermoFisher Scientific, Waltham, MA). Additionally, cDNA samples from amygdala and LH tissue were sent to the UNC Chapel Hill Advanced Analytics Core for further qPCR analysis.

Animal exclusion criteria

Animals were removed from analysis if they were found to be a significant outlier via the Grubbs test (Alpha=0.05), if a cannula misplacement (unilateral or bilateral) was found, DREADD expression was misplaced/missing, if GFP expression (as a marker of cre deletion) was misplaced or missing, or if they drank less than 0.30 g /kg in the first week. In total, 2 females and 3 males from the Gi DREADD group, and 7 females and 4 males from the control DREADD group were excluded due to no or unilateral DREADD expression. Additionally, 1 female receiving 6 cycles of DID was excluded from analysis of CRF mRNA expression in the amygdala and 1 female and 1 male receiving 3 cycles of DID were excluded from analysis of CRF1R mRNA expression in the amygdala due to significantly outlying data. No animals from the pharmacology experiment had misplaced cannula.

Statistical Analysis

All analyses and graphs were generated with SPSS (IBM Analytics, Armonk, New York) and GraphPad Prism (GraphPad Software, Inc. La Jolla, CA, USA). Unpaired two-tailed t-tests, or repeated measures analysis of variance (ANOVA) were used where appropriate, to assess experimental treatment effects on ethanol intake, BEC's and sucrose intake. Relative expression of mRNA was analyzed using the comparative CT ($\Delta\Delta$CT) method. Two-way ANOVAs, or Kruskal-Wallis tests for non-parametric data, were performed to examine the effects of repeated cycles of DID of mRNA expression. Bonferroni corrected t-tests and planned comparisons were used for significant ANOVA effects where appropriate. All data are reported as the mean ± standard error of the mean considered significant at $p < 0.05$ (two-tailed). For our genetic deletion experiments we

used Repeated Measures Two Way ANOVA, student's t-test and Mixed effect analysis. If main effects were present, Šídák multiple comparison post hoc test was performed, p value smaller than 0.05 was considered significant. Both unilateral and bilateral hits were included in this study. Data points were removed according to exclusion criteria when applicable, see extended methods in supplemental.

RESULTS

<u>Gi DREADD silencing of the CeA to LH pathways revealed a sex-specific reduction for ethanol drinking in males only.</u>

Fig. 1 shows a schematic of virus placement into the CeA and cannulae placement into the LH, as well as photomicrographs of virus expression in the CeA and terminal expression in the LH. **Fig. 2A** shows the timeline of manipulations in the chemogenetic experiment. Placing the *Gi* DREADD into the CeA, and activating it with CNO injections from the LH, there was a trend towards significance with treatment ($F(1,26)=3.76$, $p=0.063$), and a significant effect of sex ($F(1,26)=17.68$, $p< 0.001$) on ethanol intake. The interaction between sex and treatment also had a significant impact on ethanol consumption ($F(1,26)= 4.86$, $p=0.04$). Planned comparisons for vehicle and CNO injection groups of each sex showed that CNO injections significantly reduce binge-like ethanol drinking in males ($p=0.009$), but not females ($p=0.861$) (***Fig 2B***). Blood ethanol concentrations (BECs) showed a significant main effect for injection type ($F(1,26)= 4.114$, $p=0.053$), as well as sex ($F(1,26)= 8.19$, $p=0.008$). The interaction of sex and treatment was not a significant factor of BECs ($F(1,26)=2.444$, $p=0.130$). Again, this effect reflects a significant reduction in BEC's for males injected with CNO relative to vehicle ($p=0.021$),

and not females (p=0.736) (***Fig 2C***). For DID sucrose drinking there was no significant impact of treatment (F(1,26)=3.545, p=0.071). The main effect of sex on sucrose consumption was significant, such that females overall drank more than males (F(1,26)= 7.47, p=0.01). The interaction of sex and treatment again did not significantly affect sucrose intake (F(1,26)=0.320, p=0.576). (***Fig 2D***). **Fig 1E** shows the cannulae placement map.

In *mCherry* control DREADD study there was no change in ethanol consumption based on sex (F(1,12)=1.921, p=0.191), compound injected (F(1,12)=0.03998, p=0.8449), or an interaction of these two factors (F(1,12)=0.3613, p=0.559) as seen in **Fig 2F**. Similarly, variation in BECs was not a consequence of sex (F(1,12)=0.03295, p=0.859), CNO/Veh injections (F(1,12)=1.142, p=0.3062), or an interplay of the two (F(1,12)=1.33, p=0.2713) as seen in **Fig 2G.** Lastly, when assessing sucrose consumption no significant changes were seen when considering the main effect of sex (F(1,12)=2.488, p=0.1407), treatment (F(1,12)=0.3251, p=0.5791), or an interaction of sex by treatment (F(1,12)=0.01794, p=0.8957), shown in **Fig 2H**. **Fig 2I** shows the cannulae placement map. In summary, activation of Gi DREADD significantly reduced binge-like ethanol intake and BECs in male, but not female, mice, and CNO was without effect in control virus treated animals.

<u>Pharmacological inhibition of CRF1R in the LH blunts binge-like ethanol intake in male, but not female mice, with no such effects seen during pharmacological activation of CRF2R.</u>

To focus on the CRF circuitry within the LH, mice were injected with a CRF1R antagonist (NBI-35965) or CRF2R agonist (Ucn3) into the LH. A timeline of procedures

from the pharmacology experiments is presented in **Fig. 3A.** Relative to vehicle treatment, inhibiting CRF1 receptors in the LH resulted in reduced binge-like ethanol consumption such that the interaction between sex and treatment was significant (F(1,18)= 6.870, p=0.0173), while the main effects of sex (F(1,18)=0.0546, p=0.8179) and treatment (F(1,18)=0.001, p=0.9745) had no significant effect on ethanol consumption. Using paired samples t-test as planned comparisons to assess differences between vehicle and NBI-35965 treatments, there was a significant reduction in ethanol drinking for males treated with NBI-35965 (t(9)= 2.577, p=0.03), but not in females (t(9)= 1.510, p=0.165) (***Fig 3B***). BEC's reflect the ethanol drinking data in that only the interaction of sex by compound injection was significant (F(1,35)= 4.854, p=0.0343), while the main effect of sex (F(1,35)=0.4033, p=0.5295) and treatment condition (F(1,35)=2.079, p=0.1582) was not. Planned comparisons again show that the significant CRF1R antagonist-induced reduction in BEC's occurs in males (p=0.0263), and not females (p>0.9999) ***(Fig 3C)***. Sucrose drinking remained stable with no significant main effects of sex (F(1,18)=0.4619, p=0.5054), treatment (F(1,18)=0.4645, p=0.5042), or an interaction of these two factors (F(1,18)=0.1778, p=0.6783) ***(Fig 3D).*** **Fig. 3E** shows the cannulae placement map for the pharmacology experiment.

Next, involvement of the CRF2R was tested via the use of the selective agonist UCN3. Overall, females consumed more ethanol than males (F(1,13)=24.78, p=0.0003), but this effect was independent of whether animals received a UCN3 or vehicle microinjection (F(1,13)=0.6456, p=0.4361). Similarly, there was no significant interaction effect on ethanol consumption when considering sex and compound injected (F(1,13)=0.9005, p=0.3599) (**Fig 3F**). BEC values reflected ethanol binge-drinking

findings, such that overall females had higher BECs than males ($F(1,13)=35.89$, $p<0.0001$), while drug treatment had no overall effect on BECs ($F(1,13)=0.06185$, $p=0.8075$). An interaction of sex x treatment did also not significantly alter BECs ($F(1,13)=0.1503$, $p=0.7045$) (**Fig 3G**). Lastly, sucrose consumption did not vary based on sex ($F(1,13)=0.8839$, $p=0.3643$), or drug treatment ($F(1,13)=0.06682$, $p=0.8001$). Likewise, the interaction of sex and drug treatment did not lead to significant changes in sucrose consumption ($F(1,13)=0.6334$, $p=0.4404$) (**Fig 3H**). **Fig. 3I** shows the cannula placement for the UCN3 experiment. In summary, pharmacological experiments show the LH CRF1R signaling modulates binge-like ethanol intake in male, but not female mice which is consistent with the chemogenetic study.

<u>Repeated cycles of binge-like ethanol consumption alter CRF and CRF receptor mRNA in the amygdala, but not in the LH</u>

A timeline of experimental procedures from the qPCR experiment is presented in **Fig. 4A**. Ethanol consumption and BECs associated with the mRNA study are shown in **Fig. 4B and C**, respectively. All groups consumed equal amounts of ethanol during the final week of DID ($F(2,32)=1.079$, $p=0.352$), and no group differences in BECs following the final session of DID were observed ($F(1,20)=0.332$, $p=0.571$), ensuring that differences in mRNA expression are the result of the number of DID cycles received. A significant main effect of sex revealed that female mice consumed more ethanol than males during the final week of DID ($F(1,32)=16.605$, $p<0.001$).

The effects of binge-like ethanol intake on CRF and CRF receptor mRNA in the amygdala are presented in **Fig. 4D-E**. A two-way ANOVA revealed that CRF mRNA expression in the amygdala was significantly different between groups ($F(3,15)=3.313$,

p=0.049), where mice that received 3 cycles of DID had significantly less CRF mRNA compared to water control mice (**Fig. 4D** p=0.042). No significant main effect of sex or group by sex interaction was found (F(1,15)=0.734, p=0.405; F(3,15)=0.114, p=0.951). As seen in **Fig. 4E,** the same effects of binge-like ethanol consumption on CRF mRNA in the amygdala were observed from the qPCR data generated by the Advanced Analytics Core (Group: F(3,15)=4.377, p=0.021; Sex: F(1,15)=0.519, p=0.482; Group x sex: F(3,15)=0.057, p=0.981). Additional data (**Fig. 4F-G**) from the Advanced Analytics Core demonstrated that there was no main effect or group, sex, or group by sex interaction on CRF1R mRNA expression in the amygdala (F(3,14)=0.597, p=0.628; F(1,14)=0.137, p=0.717; F(3,14)=0.152, p=0.926), but there was an effect of binge-like ethanol consumption on CRF2R mRNA expression in the amygdala. A two-way ANOVA revealed a significant main effect of group (F(3,16)=6.336, p=0.005), and planned comparisons revealed that mice that received three cycles of DID, six cycles of DID, or six cycles of DID followed by a 24 hour period of abstinence showed greater CRF2R mRNA expression in the amygdala compared to water control mice (p=0.049, p=0.009, p=0.014, respectively). No main effect of sex or group by sex interaction was observed (F(1,16)=0.002, p=0.969; F(3,16)=0.793, p=0.516). The effects of binge-like ethanol consumption on CRF receptor mRNA in the LH are shown in **Fig. 4H-K**. We observed no main effect of group, sex, or group by sex interaction on either CRF1R or CRF2R mRNA expression in the LH (CRF1R: F(3,16)=0.669, p=0.583; F(1,16)=0.350, p=0.562; F(3,16)=1.264, p=0.320; CRF2R: F(3,16)=1.766, p=0.194; F(1,16)=0.377, p=0.548; F(3,16)=1.225, p=0.333). Additionally, no differences were observed in CRF1R or CRF2R mRNA expression in the LH when examining qPCR data produced by the Advanced

Analytics Core (CRF1R: F(3,16)=1.645, p=0.219; F(1,16)=0.198, p=0.662; F(3,16)=0.512, p=0.680; CRF2R: F(3,16)=0.668, p=0.584; F(1,16)=0.510, p=0.485; F(3,16)=1.334, p=0.298). In summary, a history of binge-like ethanol intake was associated with reduced CRF mRNA and increased CRF2R mRNA in the amygdala, with no impact on mRNA in the LH.

CeA deletion of CRF

To determine if CRF produced in the CeA plays a role in alcohol drinking, we knocked down CRF in the CeA and measured alcohol consumption in male and female mice using the DID paradigm (**Fig. 5A**). FISH was used to validate Crh deletion, and an Unpaired Student's T-Test (p=0.0127) suggests there was a significant decrease in Crh punctae in cre treated CRF floxed mice when compared to control treated floxed mice, indicating that the Crh genetic deletion model works as intended (**supplemental Fig. S2**). Male data are presented in **Fig. 5B, C, and F**, and female data are presented in **Fig. 5D, E, and G**. Given the known differences in CRF function in the CeA, we opted to analyze the male and females separately. When comparing male Control and Cre treated mice in the weekly 2-hr consumption (**Fig. 5B**), mixed-effect analysis suggests that there was a main effect of viral treatment (F(1,24)=4.438, p=0.0458), but no main effect of time (F(2,47)=0.2971, p=0.7443) or time by viral treatment (F(2,47)=2.542, p=0.0895). Šídák multiple comparison post hoc tests between male control and cre treated CRF floxed mice indicates a significant decrease in 2hr weekly average alcohol consumption only at week 1 (p=0.0167) with no significant difference in week 2 (p=0.2945) or 3 (p=0.9622). When comparing the weekly 4hr alcohol intake of male control and cre treated CRF floxed mice (**Fig. 5C**), Mixed-effect analysis suggest there was only a main effect of time

(F(1.654,38.88)=3.797, p=0.0386), with no main effect of viral treatment (F(1,24)=2.733, p=0.1113) or time by treatment (F(2,47)=0.9377, p=0.3987). Šídák multiple comparison post hoc tests between male Control and Cre treated CRF floxed mice indicates a significant decrease in 2hr weekly average alcohol consumption only at week 1 (p=0.0290) with no significant difference in week 2 (p=0.8855) or 3 (p=0.5381). Notably when comparing cumulative daily drinking of male Control and Cre treated CRF floxed mice (**Fig. 5F**), two-way repeated measures ANOVA suggest there was a main effect of time (F(1.177,25.89)=133.8, p<0.0001), viral treatment (F(1,22)=4.946, P=0.0367) and time by treatment (F(11,242)=2.031, p=0.0263). While there was an overall reduction in drinking in the male cre treated CRF floxed mice, Šídák multiple comparisons post-hoc tests indicate that this was not statistically significant at any of the 12 days. When comparing female Control and Cre treated CRF floxed mice 2hr weekly average alcohol consumption (**Fig. 5D**), mixed-effect analysis shows there was a main effect of viral treatment (F(1,25)=4.880, p=0.0366), but no main effect of time (F(1.821,44.62)=0.9119, p=0.4009) or time by treatment (F(2,49)=0.6031, p=0.5511). As opposed to male mice, Šídák multiple comparisons post-hoc tests indicate a significant increase in alcohol intake between Control and Cre treated female mice in week 1 (p=0.0328), but not week 2 (p=0.1085) and 3 (p=0.1551). Furthermore, when comparing female Control and Cre treated CRF floxed mice weekly 4hr alcohol intake (**Fig. 5E**), mixed-effect analysis show there was a main effect of viral treatment (F(1,25)=4.471, p=0.0446), and no main effect for time (F(1.862,45.61)=0.1569, p=0.8407) and time by viral treatment (F(2,49)=0.1903, p=0.8273). Despite the overall increase in alcohol intake in female Cre mice, Šídák multiple comparisons post-hoc tests indicate no significant differences between Control

and Cre females in weeks 1 ($p=0.4619$), 2 ($p=0.1874$) or 3 ($p=0.0772$). Notably, when comparing cumulative daily drinking of female Control and Cre treated CRF floxed mice (**Fig. 5G**), two-way repeated measures ANOVA show there was a main effect in viral treatment ($F(1,23)=9.228$, $p=0.0058$), time $F(1.212,27.88)=208.5$, $p<0.0001$) and time by treatment ($F(11,253)=7.396$, $p<0.0001$). Šídák multiple comparison post-hoc tests indicate this increase in alcohol intake was statistically different in days 7 ($p=0.0429$), 8 ($p=0.0393$), 9 ($p=0.0499$), and 10 ($p=0.0482$). Interestingly, we conducted an exploratory analysis comparing deletion of CRF in male and female mice and found in all drinking assessments, there was a sex x virus interaction, supporting potential differential effects of deletion. **Supplemental Fig.S3** illustrates the representative location where maximum virus was localized per animal for the Control and Cre CRF floxed male and female mice.

<u>CeA deletion of CRF1R</u>

We wanted to determine if local CeA CRF1R plays a role in binge-like alcohol drinking (**Fig. 6A**). FISH was used to validate Crhr1 deletion, and an unpaired student's T-test ($p=0.0166$) indicates that there was a significant decrease in Crhr1 punctae in Cre treated CRF1R floxed mice when compared to Control treated CRF1R floxed mice (**supplemental Fig. S4**). This indicates that the crhr1 genetic deletion model works as intended. Male data are presented in **Fig. 6B, C, and F**, and female data are presented in **Fig. 6D, E, and G**. When comparing both male and female Control and Cre treated CRF1R floxed mice, there was no main effect of viral treatment, time, and time by viral treatment at the 2hr weekly average alcohol consumption, 4hr weekly alcohol consumption, and cumulative alcohol consumption (**Fig. 6B-G**). This suggests that CRF1R synthesized in CeA neurons does not play a critical role in binge-like alcohol

consumption. **Supplemental Fig. S5** illustrates the representative location where maximum virus was localized per animal for the Control and Cre CRF1R floxed male and female mice. . In summary, genetic deletion of CRF from the CeA was associated with reduced ethanol intake in male mice but increased ethanol intake in female mice, while deletion of CRF1R from the CeA was without effect.

References

1. Pleil KE, Rinker JA, Lowery-Gionta EG, Mazzone CM, McCall NM, Kendra AM, et al. (2015): NPY Signaling Inhibits Extended Amygdala CRF Neurons to Suppress Binge Alcohol Drinking. *Nature neuroscience*. 18:545-545.
2. Sanford CA, Soden ME, Baird MA, Miller SM, Schulkin J, Palmiter RD, et al. (2017): A Central Amygdala CRF Circuit Facilitates Learning about Weak Threats. *Neuron*. 93:164-178.
3. MacLaren DA, Browne RW, Shaw JK, Krishnan Radhakrishnan S, Khare P, Espana RA, et al. (2016): Clozapine N-Oxide Administration Produces Behavioral Effects in Long-Evans Rats: Implications for Designing DREADD Experiments. *eNeuro*.
4. Andreoli M, Marketkar T, Dimitrov E (2017): Contribution of amygdala CRF neurons to chronic pain. *Experimental Neurology*. 298:1-12.
5. Companion MA, Gonzalez DA, Robinson SL, Herman MA, Thiele TE (2022): Lateral habenula-projecting central amygdala circuits expressing GABA and NPY Y1 receptor modulate binge-like ethanol intake in mice. *Addiction neuroscience*.100019.
6. Marshall SA, Robinson SL, Ebert SE, Companion MA, Thiele TE (2022): Chemogenetic Inhibition of Corticotropin-Releasing Factor Neurons in the Central Amygdala Alters Binge-Like Ethanol Consumption in Male Mice. *Behavioral Neuroscience*. 136:541-550.
7. Thiele TE, Navarro M (2014): "Drinking in the dark" (DID) procedures: A model of binge-like ethanol drinking in non-dependent mice. Elsevier Inc., pp 235-241.
8. Burnham NW, Chaimowitz CN, Vis CC, Segantine Dornellas AP, Navarro M, Thiele TE (2021): Lateral hypothalamus-projecting noradrenergic locus coeruleus pathway modulates binge-like ethanol drinking in male and female TH-ires-cre mice. *Neuropharmacology*. 196:108702-108702.
9. Dornellas APS, Burnham NW, Luhn KL, Petruzzi MV, Thiele TE, Navarro M (2021): Activation of locus coeruleus to rostromedial tegmental nucleus (RMTg) noradrenergic pathway blunts binge-like ethanol drinking and induces aversive responses in mice. *Neuropharmacology*. 199:108797-108797.
10. Robinson SL, Bendrath SC, Yates EM, Thiele TE (2023): Basolateral amygdala neuropeptide Y system modulates binge ethanol consumption. *Neuropsychopharmacology*. 49:690-698.
11. Bloodgood DW, Hardaway JA, Stanhope CM, Pati D, Pina MM, Neira S, et al. (2021): Kappa opioid receptor and dynorphin signaling in the central amygdala regulates alcohol intake. *Molecular psychiatry*. 26:2187-2199.
12. Barkell GA, Parekh SV, Paniccia JE, Martin AJ, Reissner KJ, Knapp DJ, et al. (2022): Chronic ethanol consumption exacerbates future stress-enhanced fear learning, an effect mediated by dorsal hippocampal astrocytes. *Alcoholism, clinical and experimental research*. 46:2177-2190.
13. Paniccia JE, Lebonville CL, Jones ME, Parekh SV, Fuchs RA, Lysle DT (2018): Dorsal hippocampal neural immune signaling regulates heroin-conditioned immunomodulation but not heroin-conditioned place preference. *Brain, behavior, and immunity*. 73:698-707.
